# Eosinophilic Myocarditis Resulting in Ventricular Tachycardia Storm

**DOI:** 10.7759/cureus.56779

**Published:** 2024-03-23

**Authors:** Dylan Hengst, Daniel Kandah, Ravinder Dervesh, Michael Ellerman, Justin Ugwu, Jennifer Goerbig-Campbell, Dwayne Campbell

**Affiliations:** 1 Internal Medicine, MercyOne Des Moines Medical Center, Des Moines, USA; 2 Cardiology, MercyOne Des Moines Medical Center, Des Moines, USA; 3 Interventional Cardiology, University of Kansas Medical Center, Kansas City, USA

**Keywords:** hypereosinophilic syndrome, slow ventricular tachycardia, heart failure with reduced ejection fraction, chronic eosinophilic leukemia, mural thrombus, fulminant necrotizing eosinophilic myocarditis, ventricular tachycardia (vt) storm

## Abstract

Eosinophilic myocarditis (EM) is a rare but potentially fatal complication of sustained eosinophilia that is characterized by eosinophilic infiltration into myocardial tissue. There are various etiologies of EM that can be classified into general categories: reactive, clonal, and idiopathic. We present a case of EM caused by chronic eosinophilic leukemia, a rare myeloproliferative neoplasm that frequently presents with sustained peripheral eosinophilia. This case displays several serious complications of EM, including recurrent ventricular tachycardia storm, cardiogenic shock, and mural thrombus formation despite anticoagulation. Diagnosis of EM can be difficult as formal diagnosis requires an endomyocardial biopsy. Once EM is suspected, identifying the underlying etiology of eosinophilia is critical for timely implementation of disease-specific therapy.

## Introduction

Eosinophilic myocarditis (EM) is a rare but potentially fatal complication of sustained eosinophilia that is characterized by eosinophilic infiltration into myocardial tissue. Firm data on the epidemiology of EM specifically is not available. It is known to be more prevalent in populations affected by hypereosinophilic syndrome (HES), which is rare in and of itself, with an age-adjusted incidence of 0.036 per 100,000 patients [1]. The diagnosis of EM can be difficult to make, as formal diagnosis requires histologic visualization of eosinophil infiltration into the myocardium on tissue biopsy [2]. Additionally, the signs and symptoms of EM are not unique when compared to other commonly seen etiologies of cardiac disease and dysfunction. Presenting symptoms of EM have been reported as new or worsening heart failure, cardiac arrhythmias, or acute coronary syndrome without culprit lesion identified on angiography [1]. EM can, in severe cases, show cardiac death as the initial presentation. It is not uncommon for the diagnosis to be made at the time of autopsy [3]. Given the severity of illness frequently seen in these patients and difficulties associated with obtaining a myocardial biopsy, a presumptive diagnosis of EM is often made using clinical evaluation and non-invasive imaging modalities, the gold standard of which is cardiac magnetic resonance imaging [1,3]. Once the diagnosis is confirmed or suspected, an underlying etiology of the eosinophilia must be identified in order to initiate effective treatment [1].

## Case presentation

Medical history and initial presentation

A 60-year-old male with a history of ischemic cardiomyopathy, chronic systolic heart failure with left ventricular ejection fraction of 25-30% status post (s/p) dual chamber implantable cardiac defibrillator (ICD), coronary artery disease s/p drug-eluting stent (DES) to the left anterior descending artery nearly 20 years prior, paroxysmal atrial fibrillation (PAF) on apixaban, chronic kidney disease, hypertension, and hyperlipidemia presented as a transfer from an outside hospital for concern of EM. Two months prior to transfer, he presented to the outside facility for ventricular tachycardia (VT) storm. He underwent a coronary angiogram at that time with subsequent DES to the right coronary and right posterolateral arteries. Additionally, a left ventricular mural thrombus was discovered at that time, despite anticoagulation for PAF. Two weeks later, he was readmitted to the outside facility with recurrent VT storm and transferred to a tertiary center for VT ablation. Three weeks after his ablation, he was seen in the clinic and found to be in slow VT with rates in the 110s. He was directly admitted to the outside facility and was found to have hypereosinophilia with leukocytosis of 44,000 with 59% eosinophils. With concern for EM, he was transferred to our facility for further management. At the time of transfer, he was on metoprolol 50mg daily, mexiletine 150mg three times per day (TID), amiodarone 200mg oral TID, and an amiodarone drip at 1mg/min.

Diagnostic workup and clinical management

On hospital day one, treatment with a high dose of prednisone 90 mg daily was initiated for idiopathic HES, with hydroxyurea 1 g twice daily (BID) added on hospital day three. After three days of high-dose prednisone and one day of hydroxyurea, his white count had risen to 74,600 with 64% eosinophils. Given the lack of expected reduction in response to therapy, other etiologies for eosinophilia were explored. A bone marrow biopsy, obtained earlier, resulted in a preliminary report that noted 75% eosinophils with no abnormal blasts.

On hospital day five, an antibody assay for Strongyloides stercoralis IgG resulted positive. Following discussions with infectious disease, hydroxyurea was discontinued, and steroid therapy was tapered down due to concern for parasitic infection. Treatment with Ivermectin was initiated.

**Figure 1:**
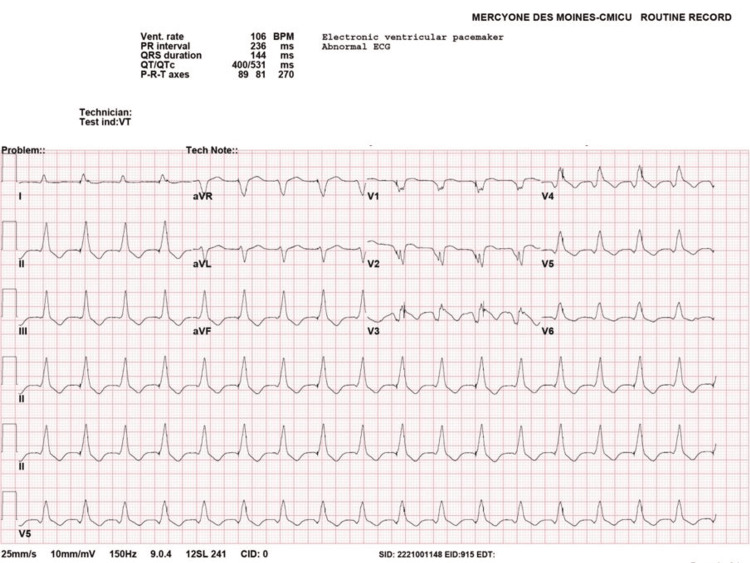
EKG demonstrating VT with a rate of 103 bpm EKG: Electrocardiogram; VT: ventricular tachycardia; BPM: beats per minute.

**Figure 2:**
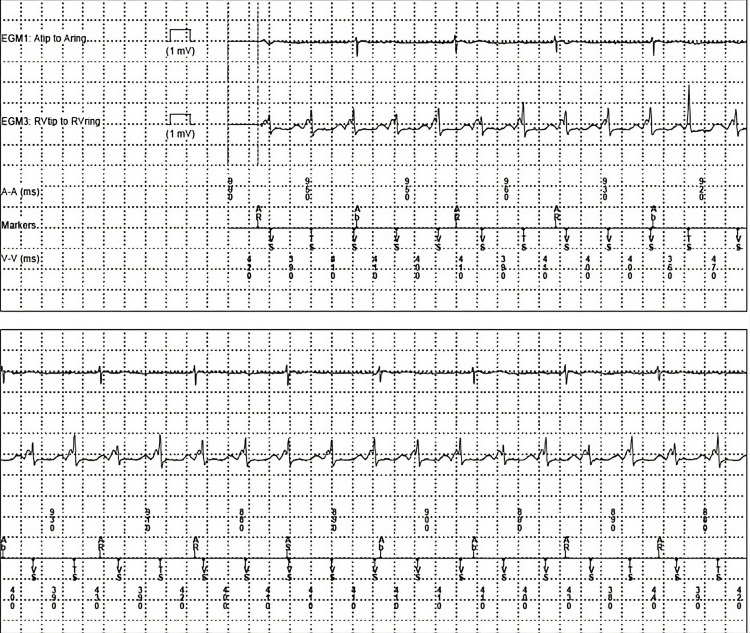
Device interrogation demonstrating VT. The atrial lead (top line) captured atrial beats at approximately 65 bpm. The ventricular lead (second line) captured ventricular beats at approximately 150 bpm VT: Ventricular tachycardia; BPM: beats per minute.

The patient had remained in sinus rhythm with first-degree atrioventricular block and premature ventricular contractions (PVCs); however, on hospital day six, the patient developed VT storm, triggering more than 20 ICD shocks. He was electively intubated and sedated to decrease adrenergic drive. Due to sedation-related hypotension, he was started on vasopressor support. He continued to have recurrent VT storm despite the up-titration of antiarrhythmic therapy. He eventually settled into a resting rhythm of slow VT with rates in the low 100s. This was confirmed on electrocardiogram (EKG) and via pacemaker interrogation, where the ventricular lead detected two beats for every one detected by the atrial lead (Figures 1, 2). 

The patient was extubated on hospital day eight, but the VT storm persisted with rates in the 140s, triggering several ICD shocks. Following this episode, the patient requested that the defibrillator function of his ICD be disabled. On hospital day ten, the flow cytometry results from the bone marrow biopsy returned, showing no signs of myeloproliferative disorder or hematologic malignancy, reinforcing earlier suspicion of reactive eosinophilia secondary to parasitic infection with strongyloides stercoralis.

**Figure 3:**
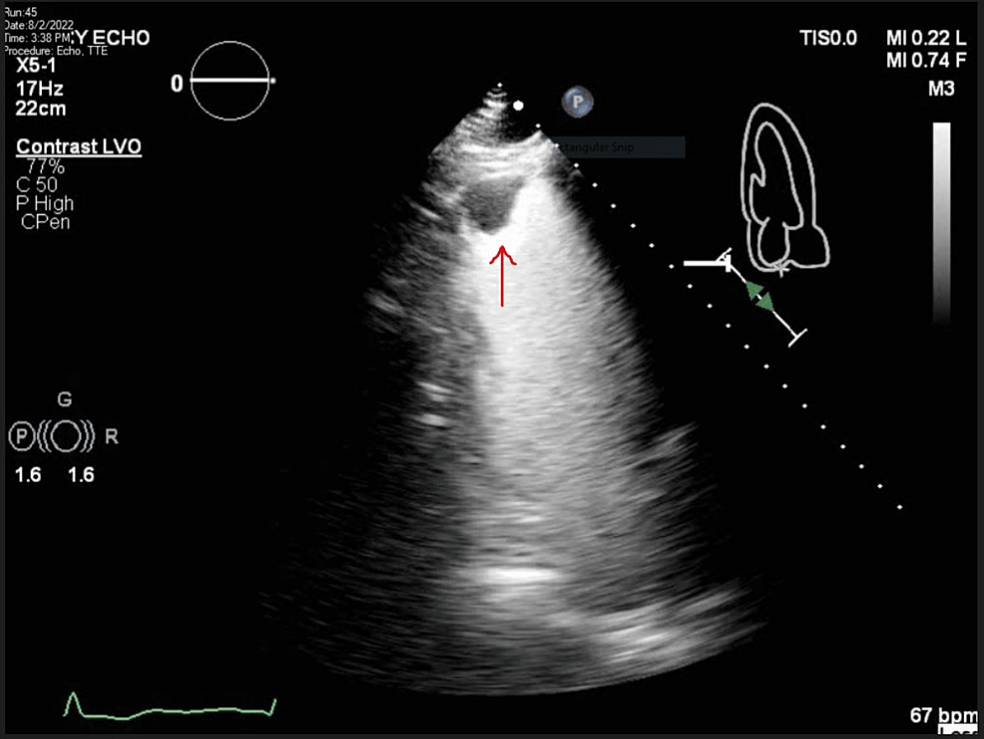
Apical two-chamber view on TTE with contrast enhancement demonstrating persistent LV apical mural thrombus TTE: Transthoracic echocardiogram; LV: left ventricle.

Over the next several days, the patient continued to experience asymptomatic runs of slow VT with rates in the 100s to 110s. Due to the continued need for vasopressor and inotropic support, up-titration of antiarrhythmics remained limited. The ventricular pacing rate was increased to 115 beats per minute (BPM) due to breakthrough VT when the pacing rate was set at 105. Following the pacing adjustments, he had no further episodes of breakthrough VT and pressor support was able to be weaned. A transthoracic echocardiogram was repeated on hospital day 17 which showed an improved ejection fraction of 35% and stable left ventricular mural thrombus (Figure 3).

The patient was discharged on day 29 with metoprolol 75 mg TID, mexiletine 150 mg TID, and amiodarone 400 mg TID. On the day of discharge, the patient had an improved but still-elevated white blood cell count of 23,000 with 71% eosinophils. 

Follow-up

Following discharge, the bone marrow biopsy was sent for further evaluation with Mayo Clinic and a diagnosis of chronic eosinophilic leukemia was made. With an underlying cause for EM now identified, treatment was adjusted to prioritize the management of chronic eosinophilic leukemia. Imatinib was started, which helped to rapidly normalize WBC and eosinophil levels but contributed to difficulty maintaining appropriate volume status as heart failure and anti-arrhythmic medications were titrated. Anti-tachycardia pacing rates of his pacemaker have been maintained at 100 beats per minute due to breakthrough VT with rates below 100. Approximately six months after discharge he continues to have persistent cardiac symptoms but has recovered much of his functional status and physical endurance. 

## Discussion

EM is a rare but potentially fatal complication of sustained eosinophilia that is characterized by eosinophilic infiltration into myocardial tissue [1]. Pathologic progression of EM is classically described in three stages: necrotic, thrombotic, and fibrotic [1-3]. The necrotic phase can present with signs and symptoms of acute heart failure, and arrhythmia and can result in cardiac death. In the thrombotic phase, a major basic protein released from eosinophils induces platelet activation and promotes a hypercoagulable state [3]. Scarring and fibrosis of the myocardium define the fibrotic phase and can contribute to long-term sequelae of cardiac disease, heart failure, or restrictive cardiomyopathy [1,2]. The resulting damage and inflammation can lead to arrhythmias ranging from isolated PVCs to VT storm, as in this case [4,5]. 

Identifying EM requires a high index of clinical suspicion. Symptoms can vary and include new or worsening heart failure, arrhythmias, and acute coronary syndrome without an obstructing lesion [1]. Once suspected, confirming the presence of EM can be difficult as formal diagnosis requires visualization of eosinophilic infiltration into the myocardium on histology. The gold standard of endomyocardial biopsy is invasive and can have a sensitivity as low as 50% [3]. 

Non-invasive imaging modalities can be used to assist in the diagnosis of EM when endomyocardial biopsy is not possible. Cardiac magnetic resonance is considered the gold standard of non-invasive imaging for evaluating myocarditis [2]. Eosinophilic myocarditis has been associated with a pattern of subendocardial late gadolinium enhancement, which can be patchy or diffuse and is not restricted to a single coronary territory [1]. Additionally, the presence of left ventricular systolic dysfunction and pericardial effusion supports the diagnosis of myocarditis [1,2]. Transthoracic echocardiography is another useful tool to aid in the diagnosis of EM and is often a first-line imaging modality to evaluate a patient with cardiac symptoms. Although there are no characteristic echocardiographic findings to specifically define EM, echocardiography allows for the assessment of cardiac function and possible complications of EM [2]. 

Polymorphic PVCs are not specific to EM and can occur in a number of conditions, including myocarditis, cardiac ischemia, and cardiac sarcoidosis to name a few. The presence of polymorphic PVCs should, however, raise concern for the presence of inflammatory, infiltrative, or ischemic processes in the myocardium [5]. PVCs with high-risk features, including frequent, polymorphic, wider QRS duration, worsening ectopy with stress or activity, or a short coupling interval should be paid particular attention as they have been associated with worse clinical outcomes. Polymorphic PVCs in particular have been shown to be associated with acute myocarditis [5].

While diagnostic workup is being pursued, it is essential to manage symptoms and provide circulatory support. While the specific treatment of EM varies based on the underlying etiology, the cardiovascular complications of EM are managed according to existing clinical guidelines [3]. These patients should be closely monitored for signs of circulatory collapse and supported with vasopressor and/or inotropic agents as needed. In the event of continued decline, there should be a low threshold for initiation of advanced mechanical circulatory support while improvements in electrical stability of the heart are achieved [5].

Treating the etiology of EM is key; these etiologies are classified into reactive, clonal, or idiopathic causes [1,6]. Reactive causes of EM, such as allergies, infections, or drug reactions should be assessed immediately [2]. Clonal work-up often includes bone marrow biopsy and may indicate further evaluation with flow cytometry, fluorescent in situ hybridization, or other cytogenetic testing [7]. Once reactive and clonal causes have been ruled out, the etiology should be considered idiopathic. In this case, the etiology was determined to be chronic eosinophilic leukemia, a rare myeloproliferative with a high response rate to tyrosine kinase inhibitors, such as imatinib [1,2]. 

## Conclusions

We presented a case of EM in a patient with significant existing cardiac disease and prior interventions. His symptoms on presentation were consistent with acute coronary syndrome and exacerbation of previously diagnosed heart failure with reduced ejection fraction. The presentation in this case could have easily been attributed to his underlying cardiac disease, and it was not until a complete blood count revealed peripheral eosinophilia, nearly two months after initial presenting symptoms, that concern for EM was raised. It is crucial to maintain a high index of suspicion when diagnosing EM as presenting features can mimic those of numerous other cardiac pathologies. Once identified, management consists of addressing the underlying cause while providing support to the cardiovascular system.
